# Integrating precision medicine through evaluation of cell of origin in treatment planning for diffuse large B-cell lymphoma

**DOI:** 10.1038/s41408-019-0208-6

**Published:** 2019-05-16

**Authors:** Grzegorz S. Nowakowski, Tatyana Feldman, Lisa M. Rimsza, Jason R. Westin, Thomas E. Witzig, Pier Luigi Zinzani

**Affiliations:** 1 Division of Hematology, Mayo Clinic, Rochester, MN USA; 2 Division of Lymphoma, John Theurer Cancer Center at Hackensack University Medical Center, Hackensack, NJ USA; 3 Department of Laboratory Medicine and Pathology, Mayo Clinic, Scottsdale, AZ USA; 4 Department of Lymphoma and Myeloma, The University of Texas MD Anderson Cancer Center, Houston, TX USA; 5 Institute of Hematology, University of Bologna, Bologna, Italy

**Keywords:** Phase II trials, B-cell lymphoma

## Abstract

Precision medicine is modernizing strategies for clinical study design to help improve diagnoses guiding individualized treatment based on genetic or phenotypic characteristics that discriminate between patients with similar clinical presentations. Methodology to personalize treatment choices is being increasingly employed in clinical trials, yielding favorable correlations with improved response rates and survival. In patients with diffuse large B-cell lymphoma (DLBCL), disease characteristics and outcomes may vary widely, underscoring the importance of patient classification through identification of sensitive prognostic features. The discovery of distinct DLBCL molecular subtypes based on cell of origin (COO) is redefining the prognosis and treatment of this heterogeneous cancer. Owing to significant molecular and clinical differences between activated B-cell-like (ABC)- and germinal center B-cell-like (GCB)-DLBCL subtypes, COO identification offers opportunities to optimize treatment selection. Widespread adoption of COO classification would greatly improve treatment and prognosis; however, limitations in interlaboratory concordance between immunohistochemistry techniques, cost, and availability of gene expression profiling tools undermine universal integration in the clinical setting. With advanced methodology to determine COO in a real-world clinical setting, therapies targeted to specific subtypes are under development. The focus here is to review applications of precision medicine exemplified by COO determination in DLBCL patients.

## Introduction

Precision medicine is revolutionizing patient care by reshaping drug development and improving diagnoses to guide treatments. It is defined as individualized treatment strategies based on the genetic or phenotypic characteristics that discriminate between patients with similar clinical presentations^1^. Cancer treatment has seen significant diagnostic and therapeutic advances from the increasing clinical implementation of personalized medicine^2^. Individual molecular and genetic profiling have augmented traditional cancer classification methods leading to more effective treatment strategies. For example, the use of molecular profiling has led to the development of breakthrough therapies including trastuzumab for HER2 positive breast cancer^3^ and vemurafenib for BRAF V600E positive melanoma^4,5^.

Precision methods are increasingly employed in clinical trials and have yielded favorable outcomes. A recent meta-analysis of phase II clinical trials of single-agent therapies across cancer types from 2010–2012 revealed that personalized approaches significantly correlated with improved response rates, progression free survival (PFS), and overall survival (OS)^6^. Likewise, analysis of clinical trials across cancer types that led to FDA drug approvals between 1998 and 2013 revealed that use of personalized approaches correlated with a higher relative response rate ratio and longer PFS, with a trend toward prolonged OS^7^. These meta-analyses underscore the potential for employment of precision medicine to guide cancer treatment.

A relevant representation of precision medicine in B-cell malignancies is the classification of diffuse large B-cell lymphoma (DLBCL) based on cell of origin (COO). The COO concept used to classify lymphoma defines every lymphoid cancer by the most related non-malignant cell type based on clinical, phenotypic, or genetic characteristics^8^. Although more than 50% of DLBCL patients can be cured by currently available chemoimmunotherapy regimens, the clinical outcome of DLBCL is highly varied, underscoring the importance of classification that can lead to effective personalized treatment^8^. The focus of this review is to summarize the current methods and clinical applications of COO determination in DLBCL.

## COO in diffuse large B-cell lymphoma

### Importance of COO determination in DLBCL

Diffuse large B-cell lymphoma is a remarkably heterogeneous disease that comprises the majority of adult B-cell lymphoma cases. Owing to this feature, prognosis determination for patients with DLBCL was historically difficult prior to the establishment of a durable classification system. Patients responded initially to chemotherapy but most were unable to achieve a sustainable remission^9^. The introduction of the immunotherapy rituximab into first-line treatment (generally combined with CHOP) for DLBCL increased OS rates, however, prognosis remains poor for patients with relapsed/refractory (R/R) DLBCL^10–12^. Gene expression profiling (GEP) of DLBCL using a specialized “Lymphochip” microarray led to the identification of distinct molecular subtypes based on COO, termed activated B-cell-like (ABC) and germinal center B-cell-like (GCB) subtypes^9^. Further efforts using GEP led to the discovery of a third heterogeneous subtype of DLBCL, termed “type 3” or “unclassifiable”, which does not express genes characteristic of either ABC- or GCB-type cells^13^.

The discovery of the ABC and GCB subtypes provided a basis for some of the observed heterogeneity of this disease. On a molecular level, both the ABC and GCB subtypes overexpress the antiapoptotic protein BCL2^14^. However, ABC-type DLBCL is specifically associated with constitutively active nuclear factor kappa B (NF-κB), often via mutations in the B-cell receptor (BCR) signaling pathway^15^. Clinically, the ABC subtype is associated with worse outcomes; population-based studies report 5-year OS rates of 35% for ABC-DLBCL patients and 60% for GCB-DLBCL patients in the prerituximab era^13^ and 3-year OS rates of ≈45% for ABC-DLBCL patients and ≈80% for GCB-DLBCL patients following first-line R-CHOP treatment^16,17^. Owing to the significant molecular and clinical differences between ABC- and GCB-DLBCL subtypes, COO identification offers opportunities to optimize treatment selection per patient needs^18^. Several targeted therapies are under investigation, including proteasome inhibitors that reduce NF-κB signaling and BCR pathway inhibitors for ABC-DLBCL^18^.

ABC- and GCB-DLBCL may also be associated with additional molecular subsets that impact their clinical relevance. Abnormalities in the *MYC* oncogene and *BCL2* are associated with worse outcomes^18–22^. Double-expressor lymphoma (DEL) occurs primarily in the ABC subtype and is associated with high *MYC* and *BCL2* expression. Alternatively, double-hit lymphoma (DHL; with concurrent translocations of *MYC* and *BCL2*) is primarily found in the GCB subtype^18^. DHL may be prognostic for worse outcomes, however, a meta-analysis of DLBCL studies found that translocations of *MYC*, *BCL2*, or *BCL6* in isolation most often reported a lack of any significant prognostic information^23^. Single-hit *MYC* rearrangement alone without evidence of *BCL2* or *BCL6* rearrangement has been associated with worse prognosis compared with patients with normal *MYC*^24,25^. The 2016 NICE guidelines recommend that the presence of *MYC*, *BCL2*, and *BCL6* rearrangements should be interpreted in the context of other prognostic factors^26^. Importantly, despite the potential for *MYC* and *BCL2* expression to be considered as a prognostic biomarker, COO remains independently associated with DLBCL outcomes with prognostic significance^21^. While DHLs defined by *MYC* and *BCL2* genetic alterations are included in the updated WHO classification system, MYC and BCL2 protein alterations could be considered prognostic indicators rather than a separate category^19^. These findings have encouraged the use of COO identification in clinical practice, which is increasingly available as a result of advances in diagnostic methodology. In 2016, the WHO classification of lymphoid neoplasms was revised to include a requirement to determine COO at diagnosis for patients with DLBCL^19^.

While the focus here is on advances in methodology to differentiate key DLBCL subtypes based on COO, the field continues to pursue more detailed examinations of genetic and mutational signatures that drive disease progression, with the goal of informing the rational design of single-agent and combination therapies^27,28^.

### Methods of determination

Although widespread adoption of COO classification would greatly improve treatment and prognosis, limitations in the GEP method used to identify the ABC and GCB subtypes prevented its integration in the clinical setting. GEP using the original Lymphochip competitive array or subsequent Affymetrix oligonucleotide microarray methods required considerable time and expense, as well as fresh-frozen biopsy tissue, which prevented concurrent analysis of morphological features^29,30^. Several practical alternatives to this technique are available, including immunohistochemistry (IHC) staining and novel GEP methods using formalin-fixed, paraffin-embedded tissues (FFPE).

IHC offers several advantages for DLBCL COO determination over GEP. This method allows for direct visualization of tumor samples, minimizing the possibility for misclassification due to the presence of nontumor tissue. Furthermore, protein levels are expected to correlate better with outcome than messenger ribonucleic acid expression^31^. However, the increased morphological resolution of IHC comes at a price of decreased subtype resolution, as this method can only distinguish the GCB subtype from non-GCB (which includes both ABC and unclassifiable subtypes). Moreover, the potential for high inter- and intra-observer and laboratory variations hinder reliable classification across laboratories^32^.

Effective IHC-based COO identification requires the use of validated sets of protein markers. To this end, several algorithms have emerged that show variable concordance with GEP (Table 1). One of the earliest attempts to classify DLBCL by IHC used markers of B-cell differentiation, including CD10 and BCL6 for GCB expression patterns and IRF4/MUM1 and CD138 expression patterns, but did not predict clinical outcome^33^. Shortly thereafter, the Hans algorithm emerged and became the most commonly used method of IHC-based COO prediction^31^. This method relies on expression of CD10, BCL6, and IRF4/MUM1 to distinguish GCB from non-GCB subtypes and has a 71% and 88% overall agreement with GEP classification for the GCB and non-GCB subtypes, respectively^31^. Though Hans algorithm concordance with GEP is high, due to the prevalence of the ABC subtype, the chances that the algorithm correctly identifies an ABC subtype patient from a given sample are proportionally lower, thus potentially limiting the ability of IHC methods to retain the prognostic significance of GEP^34^. Five-year OS rates between subtypes determined by the Hans algorithm were somewhat comparable to those found by GEP (OS rates: 76% GCB and 34% non-GCB by Hans algorithm, 60% GCB and 35% ABC by GEP)^13,31^. Additional proposed methods following the establishment of the Hans algorithm included the Muris algorithm, which assesses BCL2, CD10, and IRF4/MUM1 expression^35^, identification of LMO2 as a prognostic marker by Natkunam et al.^36^, and Nyman et al.^37^ defining the ABC subtype as having positive expression of either IRF4/MUM1 or FOXP1. The Choi algorithm added GCET1 and FOXP1 to the Hans algorithm to increase accuracy and observed a 93% concordance with GEP, as well as similar OS predictive power^38^. A key feature of the IHC methods discussed thus far is that antibody staining must proceed in a specific order, putting greater weight for subtyping on the expression of some proteins over others. The Tally algorithm proposed examining CD10 and GCET1 (positive GCB markers), and FOXP1 and IRF4/MUM1 (positive ABC markers) in any order to assign a score, using the GCB marker LM02 as a tie-breaker^39^. The Tally method was associated with 93% concordance with GEP in patients treated with R-CHOP. Although the Tally and Choi methods showed higher concordance with GEP than the Hans algorithm, technical difficulties in performing IHC for GCET1, FOXP1, and LM02 prevented their widespread adoption^31,38,39^. Lastly, the Visco–Young algorithm attempted to address these limitations by examining only three markers, CD10, BCL6, and FOXP1, and was associated with 93% concordance with GEP^40^.

**Table 1:**
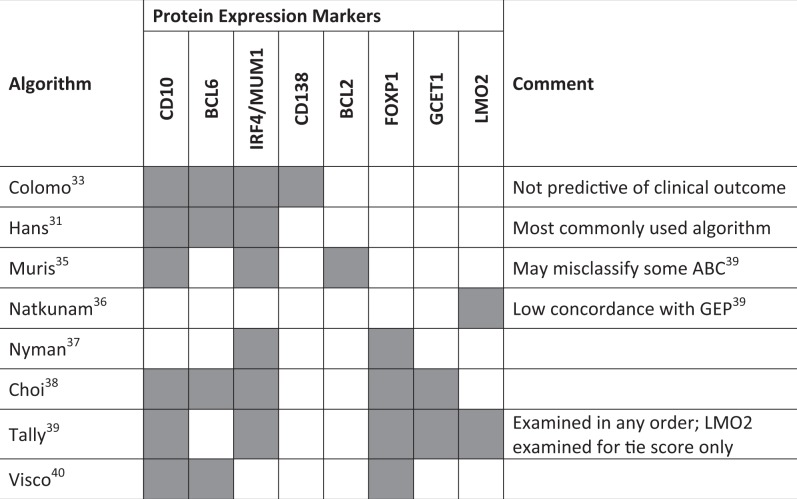
Immunohistochemistry algorithms for COO classification in DLBCL (shaded areas represent markers that are included in each algorithm)

*COO* cell of origin, *DLBCL* diffuse large B-cell lymphoma

While the ease of IHC-based methods for COO determination increases their clinical utility, success in predicting outcomes has been variable. This may be a general limitation in the way the IHC algorithms were developed; these methods were trained on retrospective population-based cohorts of patients with DLBCL, while later analysis examined cohorts of patients enrolled in clinical trials. The Hans algorithm was designed for CHOP-treated patients, and in the rituximab era was found to be less prognostic for survival^41–43^. In multiple analyses, including retrospective analysis of the RICOVER-60 trial involving 949 patients, the Hans algorithm was predictive of OS in CHOP-treated, but not R-CHOP-treated patients^41,42^. This phenomenon is not exclusive to the Hans algorithm. In a study containing 157 patients treated with rituximab-containing chemoimmunotherapy, the proportion of misclassification rates for IHC compared with GEP according to the Colomo, Hans, Muris, Choi, and Tally algorithms were 41%, 48%, 30%, 60%, and 40%, respectively. Moreover, while GEP predicted GCB and ABC classifications with 5-year OS rates of 80% and 45%, respectively, none of the IHC methods predicted OS differences between GCB and non-GCB subgroups^44^. Nonetheless, in a study of 475 patients treated with R-CHOP, the Visco–Young algorithm resulted in high concordance with GEP (93%) and strong prognostic power that matched GEP^40^. Further confounding this methodology, in the R/R setting, the Hans algorithm did not predict outcome in patients receiving ifosfamide, carboplatin, and etoposide (ICE)^45^; however, COO determined by the Hans algorithm in patients receiving rituximab plus ICE (R-ICE) or rituximab plus dexamethasone, high-dose cytarabine, and cisplatin (R-DHAP) in the CORAL study predicted a favorable response for GCB patients to R-DHAP^46^. Furthermore, there is some evidence that these methods are not classifying overlapping patient sets; an analysis comparing different IHC algorithms showed that only 4% of patients were classified as GCB and 21% as non-GCB by all 9 IHC methods tested^47^. These results indicate that IHC techniques to assess COO in DLBCL require additional refinement in the rituximab era, though IHC may be useful for certain DLBCL subtypes that remain challenging to identify, such as T-cell/histiocyte-rich large B-cell lymphoma^48^.

While the limitations of initial efforts to classify DLBCL tumors by COO using cDNA Lymphochip microarrays on flash-frozen tissue samples prevented their use for clinical purposes, advances in GEP technology over the past decade have reignited the development of new expression profiling techniques to assess COO (Table 2)^9,49,50^. Within three years of the initial establishment of COO subtypes by Alizadeh et al.^9^, a microarray platform-independent method of assigning subtype using a Bayesian predictor based on the expression over 27 key genes was developed^30^. A significant hurdle in the use of GEP to determine COO subtypes was adequate storage and processing of flash-frozen tissue samples. Accordingly, several methods were developed for use on FFPE sections. A quantitative nuclease protection assay for the expression of 12 genes that correlated significantly with COO subtype and can be applied to FFPE sections has been reported, but not widely implemented due to the requirement of an array with limited availability^51^. Subsequently, a method using the commercial Illumina platform and an algorithm identified by a DLBCL automatic classifier (DAC) correctly classified FFPE samples with a significant relationship between subtype and survival in 172 patients treated with first-line R-CHOP^52^. However, this platform has subsequently been discontinued. Based on the DAC and 20 of 27 previously-identified key genes^30^, a robust, platform-independent, subtype classifier using either fresh frozen or FFPE tissue was developed that is potentially suitable for clinical use on individual patients. Subsequently, a method was developed to probe RNA using Affymetrix technology on FFPE samples that enabled accurate disease classification with microarrays^53^. Using this classifier, comparative analysis across 10 DLBCL data sets, including 2030 patients, showed a superior separation of survival outcomes based on COO rather than other classifiers^54^. Despite its complexity compared with IHC, GEP remains a precise and valuable method for COO prediction.

**Table 2:** Comparison of methods for COO determination in DLBCL

Method	Manufacturer	Number of genes	Accuracy versus gold standard	Useful in FFPET	Interlab reproducibility	Expense
IHC	Various	1–10	++	+++	+	++
Multiplex RT-PCR	Primera Dx	1–20	++	+++	Not tested	++
qNPA	HTG-molecular	1–48	++	+++	Not tested	++
Digital array	Nanostring	10–100s	+++	+++	+++	++
Oligonucleotide array	Affymetrix	1000s	+++	++	Not tested	+++
DASL	Illumina	1000s	Not tested	+++	Not tested	+++

Reprinted from Rimsza et al.^50^, with permission from AACR

+ low; ++ moderate; +++, high

*DASL* cDNA-mediated annealing, selection, extension, and ligation, *FFPET* fresh-frozen paraffin-embedded tissue, *IHC* immunohistochemistry, *qNPA* quantitative nuclease protection assay, *RT-PCR* real-time polymerase chain reaction

**Fig. 1:**
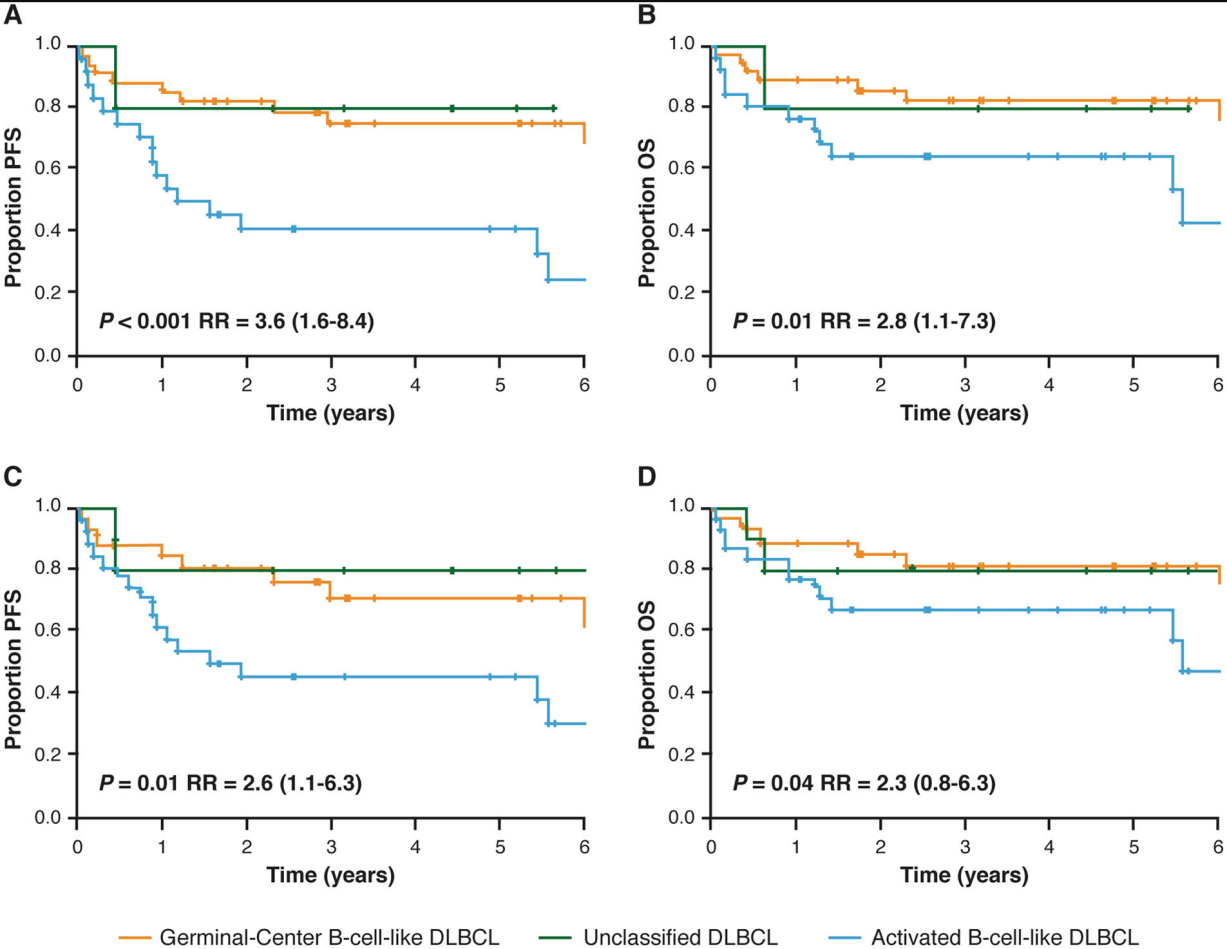
Outcomes by COO in an independent validation cohort of 68 patients receiving first-line CHOP or R-CHOP. **a** PFS by COO per Lymph2Cx, **b** OS by COO group per Lymph2Cx, **c** PFS by COO group per gold standard GEP, and **d** OS by COO per gold standard GEP. CHOP, cyclophosphamide, doxorubicin, vincristine, prednisone; COO cell of origin, GEP gene expression profiling, OS overall survival, PFS progression-free survival, R-CHOP rituximab with CHOP, RR relative risk (with 95% confidence interval). Republished with permission of Blood: a journal of the American Society of Hematology, from Scott et al.^55^; permission conveyed through Copyright Clearance Center, Inc

The current standard for predicting COO from GEP utilizes the commercially available NanoString^®^ nCounter^®^ Analysis System (NanoString Technologies, Seattle, WA, USA). The Lymph2Cx assay was developed as an economical, robust, and molecularly validated method to determine COO using GEP on FFPE tissue^55^. Examination of 93 genes previously reported to differentiate between subtypes^16^ using the NanoString^®^ nCounter^®^ Analysis System led to the identification of 20 key genes. Validation of the Lymph2Cx method in an independent cohort found a 2% error rate in COO assignment compared with 9%, 6%, and 17% error rates for the Hans, Tally, and Choi IHC algorithms, respectively^31,38,39,55^. Survival outcomes were similar to those obtained by the gold standard GEP technique by Lenz et al.^16^ (Fig. 1). Importantly, concordance between two independent laboratories employing this technique was greater than 95%^55^. Subsequent analyses using the Lymph2Cx assay examined a cohort of 344 patients with de novo DLBCL treated with R-CHOP and showed that no misclassifications were observed^21^. This method recapitulated prognostic predictions independently of International Prognostic Index score, with patients classified to the ABC subgroup having significantly inferior time to progression, PFS, disease-specific survival, and OS^21^. In addition, COO (GCB versus ABC) predicted by the Lymph2Cx assay remained prognostically-relevant independent of BCL2 and MYC protein expression status, albeit with BCL2/MYC co-expression allowing for identification of patients with poor prognosis in both COO subtypes^21,56^. The CALYM research consortium has reported a reverse transcriptase multiplex ligation-dependent probe amplification method which achieves 85% correct classification and requires only standard laboratory equipment^57^.

Currently, methods using the NanoString^®^ nCounter^®^ Analysis System are being employed to assess COO in DLBCL in clinical trials. NanoString technology was used for COO subtype identification in a subset of patients (*n* = 47) from the phase Ib/II REAL07 study, which is evaluating lenalidomide plus R-CHOP in elderly patients with DLBCL^58^. An analysis of concordance between the NanoString method and IHC using the Hans algorithm demonstrated a 6% rate of misclassification, thus demonstrating that this method was feasible for use in COO assignment in the context of a prospective clinical trial^58^. The Lymph2Cx assay is being used in real-time as a companion diagnostic in the randomized, phase III ROBUST (NCT02286062) trial testing lenalidomide/R-CHOP (R^2^-CHOP) versus placebo/R-CHOP in previously untreated ABC-DLBCL patients^59^. Preliminary evaluation of COO in an ongoing study identified ABC subtype in 33% of the patients screened, with a mean turnaround time for subtype identification of 2.25 days^60,61^. The use of the NanoString^®^ nCounter^®^ Analysis System has also begun to provide COO data in R/R DLBCL. A second method using this system was recently developed using 145 differentially expressed genes assessed in fresh frozen and FFPE biopsy samples from 18 patients with R/R DLBCL in a phase IIb fostamatinib trial^50,62^. Results were in close agreement with those reported for Lymph2Cx and 88% concordant with biopsies taken at initial diagnosis, suggesting COO stability through treatment and relapse. This level of concordance is similar to what had been reported in an analysis of the Lymph2Cx assay and the Hans algorithm in patients treated with R-CHOP^63^. Thus, GEP techniques are now sophisticated enough to be utilized in real-time for COO classification in a prospective clinical study to provide valuable data for patients with DLBCL.

## Clinical outcomes based on DLBCL subtype

With the increased availability of methods to determine COO in a real-world clinical setting, therapies targeted to specific subtypes are under development. For example, the constitutive activity of NF-κB in the ABC subtype has increased interest in agents targeting this pathway^64^. Bortezomib, ibrutinib, and lenalidomide affect NF-κB activation through different mechanisms and are under investigation in ABC-DLBCL^64–66^. Despite the success of rituximab, 40% of patients still fail first-line treatment and develop relapsed disease, and most will eventually succumb to their disease^18^. Thus, treatment strategies focusing on COO in the first-line and relapsed setting are valuable for DLBCL treatment.

### Role of COO in the first-line setting

In the first-line setting, multiple agents have been reported to improve the unfavorable outcomes associated with ABC-DLBCL by targeting overactive NF-κB signaling. Bortezomib is a proteasome inhibitor that prevents degradation of the NF-κB inhibitor IκBα^64^. Non-GCB patients treated with bortezomib plus R-CHOP experienced similar PFS and OS as GCB patients (COO determined by the Hans algorithm)^67^. The Bruton tyrosine kinase (BTK) inhibitor ibrutinib interferes with BCR signaling and downstream NF-κB activation^65^. In a phase Ib study of ibrutinib plus R-CHOP, all four non-GCB-DLBCL patients (identified by the Hans algorithm) who received any dose of ibrutinib achieved complete responses (CR)^68^. A randomized phase III study of ibrutinib in non-GCB-DLBCL patients has been initiated based on these results (NCT01855750). Lenalidomide is an immunomodulatory agent that downregulates IRF4/MUM1 and subsequently decreases BCR-dependent NF-κB activity in ABC-DLBCL cells in vitro and in tumor xenograft models^66^. A phase I trial established lenalidomide’s tolerability when administered with R-CHOP, and the combination resulted in a 100% overall response rate (ORR) in DLBCL patients^69^. Subsequently, lenalidomide was tested in two phase II studies in combination with R-CHOP (i.e., R^2^-CHOP) in patients classified by COO using the Hans algorithm^70,71^. One study of 60 DLBCL patients receiving R^2^-CHOP showed similar 2-year OS rates for GCB and non-GCB patients (75% and 83%, respectively) compared with historical controls receiving R-CHOP (no lenalidomide), where 2-year OS was significantly different in GCB and non-GCB patients (78% and 46%, respectively)^70^. The phase II REAL07 study reported similar results of 2-year PFS (71% versus 81%) and OS (88% versus 94%) rates in patients with GCB and non-GCB subtypes treated with R^2^-CHOP^71^. Overall, these studies reflect the potential impact of targeting molecular pathways based on COO specificity and the potential utility of COO determination to guide treatment choices.

### Role of COO in the relapsed setting

The prognostic significance of COO in relapsed DLBCL is less certain than in the first-line setting, as data evaluating COO in this population are more limited^72^. It is expected that there may be an increased presence of ABC/non-GCB subtypes in relapsed DLBCL due to previous studies showing worse responses for these patients to first-line therapy^45^. The role of COO in predicting survival following relapse has been investigated, although data were available in a limited number of patients. Of 985 patients who initially received anthracycline-based chemoimmunotherapy, 128 had available COO data at relapse. Of these patients, 59% had GCB and 41% had non-GCB DLBCL as defined by the Hans algorithm, directly refuting the idea that ABC/non-GCB patients are overrepresented in relapsed DLBCL, and no difference in OS was found between GCB and non-GCB patients^73^. Theoretically, it is not surprising that there are differences between lines of therapy, as first-line treatments may impact the balance or presence of certain COO subtypes in patients, with more aggressive phenotypes remaining following therapy.

The prognostic power of COO subtype analysis in R/R DLBCL patients appears to vary by treatment. The randomized phase III CORAL study compared R-ICE and R-DHAP regimens in patients in first relapse or who were refractory after first-line therapy^74^. Upon further analysis, it became evident that patients with the GCB subtype determined by the Hans algorithm had significantly improved responses to R-DHAP, while subtype was not predictive of response to R-ICE^46^. Thus, there is a clear, careful consideration of treatment combinations when evaluating the potential impact of COO subtype in DLBCL patients.

Therapies targeting overactive NF-κB signaling are being investigated in relapsed DLBCL, although preliminary results have been variable. A phase I study evaluated bortezomib combined with chemotherapy in 31 patients with R/R DLBCL who received a median of two prior therapies; patients were grouped by COO which was determined by IHC and GEP. Results of this study showed that bortezomib combined with chemotherapy provided a significantly higher ORR and OS in patients with the ABC subtype, with very little benefit derived by GCB patients^75^. The BTK inhibitor ibrutinib was evaluated in patients with DLBCL on the basis that activating mutations of the BCR pathway activate NF-κB via BTK^76^. A phase I/II trial examined single-agent ibrutinib in 80 patients with R/R DLBCL in which COO subtype was determined by GEP. Patients with the ABC subtype responded better to ibrutinib therapy, with median OS of 10.3 and 3.4 months and ORR of 37% (16% CR) and 5% (0% CR) in the ABC and GCB subtypes, respectively^76^. On the basis of these results, the phase III PHOENIX study (NCT01855750) evaluated the addition of ibrutinib to R-CHOP for patients with newly diagnosed non-GCB DLBCL. This study did not meet its primary endpoint of event-free survival, although subtype analysis is ongoing^77^.

Lastly, multiple studies have shown the benefits of lenalidomide treatment to patients with relapsed, non-GCB DLBCL. A retrospective analysis of 40 R/R DLBCL patients treated with salvage lenalidomide reported significantly higher ORR (53% versus 9%) and longer PFS (6.2 versus 1.7 months) in patients with the non-GCB compared with the GCB subtype determined by IHC using the Hans algorithm^78^. Subsequently, the DLC-001 phase II/III study of 102 patients with stem cell transplantation-ineligible, R/R DLBCL who received single-agent lenalidomide or investigator’s choice (IC, gemcitabine, rituximab, etoposide, or oxaliplatin) found that lenalidomide significantly improved PFS (13.6 weeks lenalidomide versus 7.9 weeks IC; *P* = 0.04) and numerically improved ORR (28 versus 12%; *P* = 0.08) and median OS (31 versus 24.6 weeks; *P* = 0.67)^79^. Importantly, median PFS for lenalidomide versus IC, respectively, was more improved in non-GCB subtypes (15.1 versus 7.1 weeks; *P* = 0.02) compared with GCB subtypes (10.1 versus 9.0 weeks; *P* = 0.55) determined by IHC. When COO was stratified by GEP, through an exploratory assessment, the clinical outcome was more pronounced in ABC-type patients, but did not reach clinical significance. When lenalidomide was combined with R-ICE (RICER) in a phase I/II study, no dose-limiting toxicity was encountered and response rates were favorable (60% CR and 13% PR)^80^. COO subtype was determined by IHC, and ORRs were 100% and 60% for GCB and non-GCB patients, respectively. Responding patients received autologous stem cell transplantation (ASCT) followed by lenalidomide maintenance. Few relapses occurred following ASCT, however, all were of the GCB subtype. At present, very few trials are testing the effect of targeted therapies in DEL/DHL patients. A phase II trial is currently recruiting post-stem cell transplant DHL patients to assess ibrutinib (NCT02272686). Overall, studies in the relapsed setting indicate that newer therapies for DLBCL are effective and provide a rationale for the increased investigation of the applicability and integration of COO classification.

## Discussion

The discovery of divergent COO subtypes provides a basis for gaining increasing knowledge about the clinical heterogeneity of DLBCL at a molecular level. In a real-world application of precision medicine, COO determination at diagnosis advises physicians on anticipated patient prognosis and is increasingly used to inform selection of therapy. The method used to determine COO is an important consideration, as methods based on IHC have a higher rate of misclassification versus GEP. From a retrospective perspective, misclassifications have few consequences, however, when being used to guide first-line treatment, the cost of misclassification can be significant. Thus, it is essential to continue to refine and cross-validate the methods used to determine COO clinically. Nonetheless, the ability to perform real-time COO classification has encouraged the continued evaluation of molecular-based agents including bortezomib, ibrutinib, and lenalidomide, alone or in combination, to improve the dismal prognosis for patients with higher risk DLBCL. Although promising, evidence to date is limited, particularly in the R/R setting. As ongoing and planned studies increasingly make use of GEP assays as companion diagnostics, evidence-based fine-tuning of therapies based on COO and additional pathobiological factors will help lay the groundwork for providing improved clinical benefit to patients with this aggressive disease.
